# GLABRA2, a Common Regulator for Epidermal Cell Fate Determination and Anthocyanin Biosynthesis in *Arabidopsis*

**DOI:** 10.3390/ijms20204997

**Published:** 2019-10-09

**Authors:** Siyu Chen, Shucai Wang

**Affiliations:** 1 College of Life Science, Linyi University, Linyi 276005, China; 23329915.ok@163.com; 2 Key Laboratory of Molecular Epigenetics of MOE and Institute of Genetics & Cytology, Northeast Normal University, Changchun 130024, China

**Keywords:** GLABRA2, trichome initiation, root hair formation, anthocyanin biosynthesis, MBW activator complex, *Arabidopsis*

## Abstract

Epidermal cell fate determination—including trichome initiation, root hair formation, and flavonoid and mucilage biosynthesis in *Arabidopsis* (*Arabidopsis thaliana*)—are controlled by a similar transcriptional regulatory network. In the network, it has been proposed that the MYB-bHLH-WD40 (MBW) activator complexes formed by an R2R3 MYB transcription factor, a bHLH transcription factor and the WD40-repeat protein TRANSPARENT TESTA GLABRA1 (TTG1) regulate the expression of downstream genes required for cell fate determination, flavonoid or mucilage biosynthesis, respectively. In epidermal cell fate determination and mucilage biosynthesis, the MBW activator complexes activate the expression of *GLABRA2* (*GL2*). GL2 is a homeodomain transcription factor that promotes trichome initiation in shoots, mucilage biosynthesis in seeds, and inhibits root hair formation in roots. The MBW activator complexes also activate several R3 MYB genes. The R3 MYB proteins, in turn, competing with the R2R3 MYBs for binding bHLH transcription factors, therefore inhibiting the formation of the MBW activator complexes, lead to the inhibition of trichome initiation in shoots, and promotion of root hair formation in roots. In flavonoid biosynthesis, the MBW activator complexes activate the expression of the late biosynthesis genes in the flavonoid pathway, resulting in the production of anthocyanins or proanthocyanidins. Research progress in recent years suggests that the transcriptional regulatory network that controls epidermal cell fate determination and anthocyanin biosynthesis in *Arabidopsis* is far more complicated than previously thought. In particular, more regulators of *GL2* have been identified, and GL2 has been shown to be involved in the regulation of anthocyanin biosynthesis. This review focuses on the research progress on the regulation of *GL2* expression, and the roles of GL2 in the regulation of epidermal cell fate determination and anthocyanin biosynthesis in *Arabidopsis*.

## 1. Introduction

**Figure 1:**
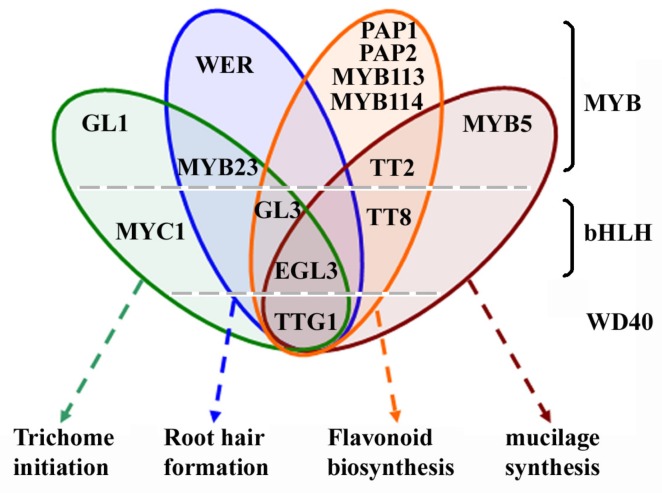
Components of the epidermal cell fate determination, flavonoid and mucilage biosynthesis regulating MBW activator complexes. Some of the components are presented in all the MBW activator complexes, some are not. The WD40-repeat protein TTG1, and the bHLH transcription factors EGL3 are involved in the regulation of epidermal cell fate determination, flavonoid and mucilage biosynthesis. The bHLH transcription factor MYC1 is involved in the regulation of trichome initiation. TT8 is involved in the regulation of flavonoid and mucilage biosynthesis. The MYB transcription factors involved in the regulation of epidermal cell fate determination, flavonoid and mucilage biosynthesis are largely different. WER is involved in the regulation of root hair formation. GL1 is involved in the regulation of trichome initiation, MYB5 is involved in the regulation of mucilage biosynthesis, and PAP1, PAP2, MYB113 and MYB114 are involved in the regulation of flavonoid biosynthesis. However, MYB23 is involved in the regulation of trichome initiation and root hair formation, whereas TT2 is involved in the regulation of flavonoid and mucilage biosynthesis.

Interplay of several different types of transcription factors, including the WD40-repeat protein TRANSPARENT TESTA GLABRA1 (TTG1) [1,2], the R2R3 MYB transcription factor GLABRA1 (GL1), MYB23 and WEREWOLF (WER) [3,4,5], the bHLH (basic helix-loop-helix) transcription factors MYC1, GLABRA3 (GL3) and ENHANCER OF GL3 (EGL3) [6,7,8,9,10] regulates epidermal cell fate determination, including trichome initiation and root hair formation in *Arabidopsis* (*Arabidopsis thaliana*). Based on the characterization of gain-, and/or loss-of-function mutants of these transcription factors genes, and protein–protein interaction assays in yeast and plant cells, it has been proposed that these transcription factors form multiple MYB-bHLH-WD40 (MBW) transcription activator complexes to regulate trichome initiation and root hair formation [6,11,12,13]. The WD40 protein TTG1, the bHLH transcription factors GL3 and EGL3 and the R2R3 MYB transcription factor MYB23 are presented in all these MBW activator complexes, but some of the R2R3 MYB transcription factors involved in the trichome initiation and root hair formation regulating MBW complexes are different, i.e., WER specifically regulates root hair formation, whereas GL1 specifically regulate trichome formation (Figure 1). Unlike GL3 and EGL3, the bHLH transcription factor MYC1 has only been reported to be involved in the regulation of trichome initiation [6].

Biosynthesis of flavonoids including anthocyanin and proanthocyanidin in *Arabidopsis* is regulated by similar MBW activator complexes (Figure 1). Some of the components in the MBW activator complexes, including the WD40-repeat protein TTG1, the bHLH transcription factors GL3 and EGL3 are the same ones involved in the regulation of trichome initiation and root hair formation, whereas others are not. An additional bHLH transcription factor, TRANSPARENT TESTA 8 (TT8) is involved in the regulation of flavonoid biosynthesis, and the R2R3 MYB transcription factors that involved in the regulation of flavonoid biosynthesis are TT2, PRODUCTION OF ANTHOCYANIN PIGMENT 1 (PAP1), PAP2, MYB113 and MYB114 [10,12,14,15,16,17,18,19,20]. It should be noted that TTG1, EGL3, TT8, and TT2 are also regulators of mucilage biosynthesis [13], but another R2R3 MYB transcription factor, MYB5, specifically regulates mucilage biosynthesis [21]. In addition to mucilage biosynthesis, MYB5 is also involved in the regulation of trichome development [22].

In the process of trichome initiation and root hair formation, the MBW activator complexes activate the expression of *GLABRA2* (*GL2*), a subfamily IV (HD-GLABRA2 group) homeodomain leucine zipper (HD-ZIP) protein gene [23,24], leading to promotion of trichome initiation in shoots, and inhibition of root hair formation in roots [11,12,13,25,26]. It should be mentioned that there are a total of four subfamilies of HD-ZIP proteins in *Arabidopsis*, and these proteins regulate multiple aspects of plant growth and development, as well as plant response to environmental stimuli [27,28].

The same MBW activator complexes can also activate the expression of single-repeat R3 MYB genes including *TRY* (*TRIPTYCHON*), *CPC* (*CAPRICE*), *ETC1* (*ENHANCER OF TRY AND CPC 1*) and *ETC3*. The R3 MYB proteins, in turn, play an lateral inhibition role by competing with R2R3 MYB transcription factors GL1, MYB23 or WER for binding bHLH proteins GL3 or EGL3, thus blocking the formation of the MBW activator complexes, resulting in inhibition of trichome initiation and promotion of root hair formation [11,12,13,25]. In the process of flavonoid biosynthesis, however, the MBW activator complexes directly regulate the expression of late biosynthesis genes in the flavonoid biosynthesis pathway including *FLAVANONE 3’-HYDROXYLASE* (*F3’H*), *DIHYDROFLAVONOL REDUCTASE* (*DFR*), *ANTHOCYANIDIN SYNTHASE*/*LEUCOANTHOCYANIDIN DIOXYGENASE* (*ANS*/*LDOX*) and *UDP-FLAVONOID GLUCOSYL TRANSFERASE* (*UFGT*), leading to the accumulation of anthocyanins [12,16,17,18,20]. Whereas activation of *BANYULS* (*BAN*) by the MBW activator complexes leading to the production of proanthocyanidins [14]. 

Remarkable achievements have been made in recent years in the dissection of the transcriptional regulatory networks controlling epidermal cell fate determination and anthocyanin biosynthesis in *Arabidopsis*. In particular, GL2, the central regulator for epidermal cell fate determination has also been shown to be involved in the regulation of anthocyanin biosynthesis, and more regulators of *GL2* have been identified. Focus of the present review is on the regulation of *GL2*, and the roles of GL2 in the regulation of epidermal cell fate determination and anthocyanin biosynthesis.

## 2. Regulation of *GL2*

**Figure 2:**
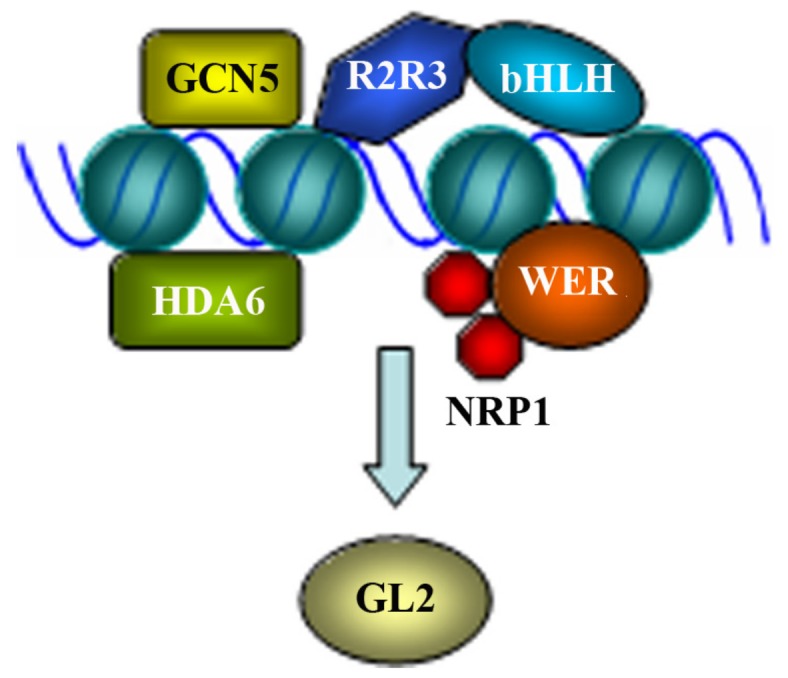
Regulation of *GL2* expression. Several different regulators are involved in the regulation of *GL2*, and the expression of *GL2* may be regulated at different ways. (i) Interaction of R2R3 MYB protein GL1 or WER and bHLH protein GL3 or EGL3 are required and sufficient to activate *GL2*, and concurrent binding of the R2R3 MYB and bHLH proteins to the promoter region of *GL2* is required for the activation. (ii) Dimer of histone chaperone NAP1-RELATED PROTEIN1 (NRP1) interacts with WER and bind to the promoter region of *GL2* to regulate its expression. NRP2 is also involved in the regulation of *GL2*. (iii) Histone deacetylase HDA6 binds directly to the promoter region of *GL2* to regulate its expression. (iv) Histone acetyltransferase GCN5 binds directly to the promoter region of *GL2* to regulate its expression. It is unclear if HDA6 and GCN5 regulated expression of *GL2* is depend on the R2R3 MYB proteins GL1 or WER and the bHLH proteins GL3 or EGL3, and if the regulators function in sequential to regulate the expression of *GL2*.

As mentioned above, the MBW activator complexes formed by the WD40-repeat protein TTG1, the bHLH transcription factors GL3 or EGL3, and the R2R3 MYB transcription factors GL1, MYB23 or WER regulate the expression of *GL2* [23,24]. In addition to these transcription factors, epigenetic modification of the chromosome by histone chaperones, histone deacetylase and histone acetyltransferase is involved in the regulation of *GL2* expression (Figure 2).

### 2.1. Transcriptional Regulation of GL2 by Transcription Factors

In *Arabidopsis*, it has been proposed that the MBW activator complexes formed by the WD40 protein TTG1, the R2R3 MYB transcription factor GL1 or MYB23, and the bHLH transcription GL3 or EGL3 regulate trichome initiation [9,13,25,26,27,28,29,30,31,32,33], whereas MBW activator complexes formed by the WD40 protein TTG1, the R2R3 MYB transcription factor WER, and the R2R3 MYB transcription factor GL3 or EGL3 regulate root hair formation [4,13,30,31,32,33,34,35,36]. Although the R2R3 MYB transcription factors in these MBW activator complexes are different, the MBW activator complexes regulate the expression of the same homeodomain protein gene, *GL2* [11,12,25]. Physically interaction of GL3/EGL3 with TTG1 and GL1 respectively, has been demonstrated in both yeast and plant cells [9,10,37,38]. The regulation of *GL2* by the MBW activator complexes is supported by several different lines of evidence. First, the expression of the *GL2* reporter gene *P_GL2_:GUS* was reduced in the *ttg1*, *gl1* and *gl3* mutants [7,39]. Second, ectopic expression of *GL1* or *R*, a *GL3* homologous gene in maize, in the *ttg1* mutants ectopically activated *GL2* expression [39]. Third, both GL1 and GL3 were found to bind to the promoter region of *GL2 in Chromatin Immunoprecipitation* (ChIP) assays, indicating that *GL2* is a directly target of GL1 and GL3 [38,40]. 

Transient transfection assays in Arabidopsis mesophyll protoplasts, however, have shed new lights to the regulation of *GL2* expression. Consistent with the proposal that *GL2* is activated by the MBW activator complexes, transfection of TTG1, GL1, WER, GL3, or EGL3 alone into *Arabidopsis* protoplasts failed to activate the expression of the endogenous *GL2* as detected by RT-PCR, and the expression of the *P_GL2_:GUS* reporter gene as detected by GUS activity assays [41]. On the other hand, co-transfection of GL1 or WER and GL3 activated both endogenous *GL2* and the *P_GL2_:GUS* reporter gene [41]. In agreement with this, overexpression of a GL1GL3 fusion protein activated the expression of the *P_GL2_:GUS* reporter gene in the stable transformed transgenic plants [42]. Co-transfection of a GL1 or WER and GL3 or EGL3 also activated some of the R3 MYB genes, including *TRY*, *CPC*, *ETC1* and *ETC3*, but not *TRICHOMELESS1* (*TCL1*), *TCL2*, and *ETC2* [43,44]. These results suggest that a complex formed by a R2R3 MYB transcription factor and a bHLH transcription factor is required and is sufficient to activate the expression of both *GL2* (Figure 2) and some of the R3 MYB genes. Consistent with this, all the DNA fragments in the *GL2* promoter region that can drive the proper expression of the *GUS* reporter gene contained at least one putative MYB-binding element and one putative bHLH binding-element [39,41]. In addition, previously experiments have shown that ectopic expression of *GL1* or a *GL3* homologous gene *R* in the *ttg1* mutants ectopically activated *GL2* [39], which also indicated that TTG1 is not required for the activation of *GL2*. However, a recent study reported that interaction of TTG1 with TT2 is required for the proper expression of *GL2* [45], suggesting that TTG1 may still play a role in the regulation of *GL2*.

By using transient transfection assays in protoplast with truncated protein, Wang and Chen [41] further showed that both the R2 domain in GL1 and the C-terminal domain in GL3 are required for the binding of *GL2* promoter, suggesting that a concurrent binding of GL1 and GL3 may be required for the activation of *GL2*. The R3 domain, on the other hand, has been shown to be required for the interaction of MYBs with bHLH transcription factors in yeast two hybridization assays [37]. Detailed analysis have identified [D/E]L×2[R/K]×3L×6L×3R in the R3 domain, as a conserved amino acid signature required for the interaction of MYBs with bHLH transcription factors [37]. As a matter of fact, this amino acid signature is conserved in GL1, MYB23, WER and all the *Arabidopsis* R3 MYB proteins [13,42,43]. In other plants such as poplar and rice, the [D/E]L×2[R/K]×3L×6L×3R amino acid signature is not fully conserved in the R3 MYBs. However, ectopic expression any of these R3 MYB genes resulted in inhibition of trichome initiation, and down regulation of *GL2* [46,47]. Assays in transfected protoplast also showed that all the rice and poplar R3 MYBs interacted with GL3/EGL3, indicating that substitution of at least some of the conserved amino acid residues in the [D/E]L×2[R/K]×3L×6L×3R amino acid signature does not affect the interaction of R3 MYB with bHLH transcription factors [46,47]. 

Characterization of *gl1-S92F*, a loss-of-function mutant shed additional lights into the regulation of *GL2* [42]. In the *gl1-S92F* mutant, a single nucleotide mutation in the *GL1* gene resulted in a substitution of the Ser92 in the [D/E]L×2[R/K]×3L×6L×3R amino acid signature in the R3 domain of GL1 with a Phe (S92F). Protoplast transient transfection assays indicate that the interaction of GL1 and GL3 was not affected by the S92F amino acid substitution, however, contransfection of GL1-S92F and GL3 failed to activate the expression of *GL2*, and expression of *GL2 in the gl1-S92F* mutant was reduced when compared with that in the Col wild-type plants [42]. These results suggest that in addition to bind bHLH transcription factors, the R3 domain in GL1 is involved in the binding of GL1 to the promoter of *GL2*. 

### 2.2. Regulation of GL2 by Epigenetic Modification of Chromosome

Epigenetic modification is involved in the regulation of *GL2* expression (Figure 2). NAP1 (NUCLEOSOME ASSEMBLY PROTEIN1), a conserved nucleosome assembly protein in plants and animals acts as a histone chaperone. NAP1-RELATED PROTEIN1 (NRP1) and NRP2, two NAP1 homologues in *Arabidopsis* are involved in the regulation of root hair formation, as increased numbers of root hairs was observed in the *nrp1-1 nrp2-1* double mutant [48]. Expression analysis of genes involved in the regulation of root hair formation showed a decreased expression level of *GL2*, suggesting that NRP1 and NRP2 are involved in the regulation of *GL2* [48]. 

Indeed, it was soon confirmed that NRP1 is directly involved in the regulation of *GL2* [49]. By using ChIP-PCR analysis, Zhu et al. [49] found that NRP1 was enriched at the promoter region of *GL2* in wild type plants, but not the wer mutants, indicating that *GL2* is a direct target gene of NRP1, and the enrichment of NRP1 at the promoter region of *GL2* is dependent on WER. Protein–protein interaction assays in both vitro and in vivo showed that NRP1 was able to form a dimer via its N-terminal α-helix, and interacted with WER [49]. By comparing histone status and nucleosome density at *GL2* promoter in the wild type and *nrp1-1 nrp2-1* mutant plants, the authors further showed that the NRP proteins were able to promote histone release and decrease nucleosome density, thereby enables WER to form a stable complex with its target DNA [49]. 

Ectopic hair cells in the nonhair positions of root epidermis were also observed in *hda6*, a loss-of-function mutant of *HISTONE DEACETYLASE6* (*HDA6*) [50]. On the other hand, increased leaf trichome density was observed in *gcn5*, a loss-of-function mutant of histone acetyltransferase gene *GENERAL CONTROL NON-REPRESSED PROTEIN5* (*GCN5*) [51]. Expression level of *GL2* was increased in the *hda6* mutants, but decreased in the *gcn5* mutants [50,51]. Consistent with this, both HDA6 and GCN5 have been found to bind directly to the promoter regions of *GL2*, and acetylation of histone H3 on the promoter regions of GL2 was increased in the *hda6* mutant, but decrease in the *gcn5* mutants [50,51]. These evidence suggest that HDA6 and GCN5 directly regulate the expression of *GL2* by affecting the histone acetylation levels of the promoter regions of *GL2*, provided evidence that epigenetic modification of chromosome is involved in the regulation of *GL2* expression.

## 3. Roles of GL2 in Regulating Epidermal Cell Fate Determination and Anthocyanin Biosynthesis

### 3.1. Regulation of Trichome Initiation and Root Hair Formation

GL2 was first identified as a regulator for trichome outgrowth, but not for trichome initiation [23]. This was further supported by the observation that the expression pattern of the *P_GL2_:GUS* reporter gene in the *gl2* mutant was largely similar to that in the wild-type plants [39]. Later on, it was found that the *gl2* mutant produces more root hairs, indicating that GL2 is required for the regulation of root hair formation [24]. Based on the characterization of other trichome and/or root hair related mutants, including the WD40-repeat gene mutant *ttg1* [1,2], the R2R3 gene mutants *gl1* and *wer* [3,4,7,8], the bHLH gene mutants *gl3* and *egl3* [9,10], and the R3 MYB gene mutants *try*, *cpc*, *etc1*, *etc2*, *etc3*, and *tcl1* [52,53,54,55,56,57,58,59], and the analyses of protein–protein interaction in yeast cells [10,37], it was then proposed that the expression of both *GL2* and the R3 MYB genes can be activated by MBW activator complexes, and work downstream of the MBW activator complexes to regulate trichome initiation and root hair formation [11,12,13,25]. 

Even though available evidence support that both GL2 and R3 MYBs function downstream of a MBW activator complex to regulate trichome initiation and root hair formation [11,13,25,26,32,33], some other studies suggest that the roles of GL2 in the transcription factor regulatory network that regulating trichome initiation and root hair formation is far more complicated than previously thought. 

First, more feedback loops have been identified. Khosla et al [60] found that GL2 was able to activate the expression of *MYB23*, a functional homolog of *GL1* [5], thus activating a positive feedback loop to regulate trichome initiation. MYB23 has also been shown to be a functional homolog of WER, and to be able to induce its own expression, thus also provides a positive feedback loop in regulating root hair formation [61]. Wang et al. [59] found that, in addition to compete with GL1 for binding GL3 or EGL3, blocking the formation of the complexes required for the activation of *GL2*, TCL1 was able to suppress the expression of *GL1* by binding directly to its promoter region, added an additional negative feedback loop for the regulation of trichome initiation. 

Second, the regulation of R3 MYB genes, antagonistic genes of *GL2* in the regulation of trichome initiation and root hair formation, is more complicated than previously thought too. For example, it has been reported that the MYB complexes can only regulate the expression of some of the R3 MYB genes including *TRY*, *CPC*, *ETC1* and *ETC3*, but not *ETC2*, *TCL1* and *TCL2* [43], and both the *miR156* directed SQUAMOSA PROMOTER BINDING PROTEIN LIKE 9 (SPL9) and the membrane-associated NAM, ATAF1/2, and CUC (NAC) transcription factor NTM1-LIKE8 (NTL8) directly regulate the expression of *TCL1* and *TRY* [62,63]. However, SPL9 and NTL8 did interact with each other in both yeast and plant cells, indicating that they function in different pathways to regulate the expression of *TCL1* and *TRY* [63]. Considering that SPL9 alone can not activate the reporter gene in protoplast, it is very likely that SPL9 may interact with other proteins to regulate the expression of *TCL1* and *TRY* [63]. In addition, Pesch et al. [64] found that TTG1 and GL1 were able to compete for binding GL3, thus differently regulating the expression of R3 MYB genes *TRY* and *CPC*. However, co-transfection of TTG1 and GL3 failed to activate the expression of *GL2* in plant cells [43]. More recently, Li et al. [45] reported that TTG1 is able to interact with TT2 to regulate the expression of *GL2*. Considering that these studies mainly relied on fusion proteins and assays in yeast or protoplast cells [43,45,64], some of the results may need to be further examined. 

Third, analysis of double and higher order mutants between *gl2* and single-repeat R3 MYB gene mutants suggest that GL2 may play an essential role in regulating root hair formation but not trichome formation, i.e., GL2 is required for the inhibition of root hair formation, but may not be absolutely required for the promotion of trichome initiation in *Arabidopsis* [65], as mutation of R3 MYB genes in the *gl2* mutant background produced trichomes, but did not affect the root hair phenotype of *gl2* mutant [65]. It should be noted that these results were consistent with initial observation that GL2 functions as a regulator for trichome outgrowth, but not for trichome initiation [23].

The requirement for GL2 for the inhibition of root hair formation was further confirmed by Lin et al. [66]. They found that GL2 was able to directly suppress the expression *DEFECTIVE6* (*RHD6*), *RHD6-LIKE1* (*RSL1*), *RSL2*, LjRHL1-LIKE1 (*LRL1*) and *LRL2*, a few bHLH transcription factor genes that have previously been reported to be required for root hair formation, as root hair formation was inhibited in loss-of-function mutants of these genes [67,68,69,70,71]. In addition, Wu and Citovsky found that the plant specific proteins GL2-interacting repressor 1 (GIR1) and GIR2 interacted with GL2, and are involved in the regulation of root hair formation [72], suggest that GL2 may also function in complex with other regulators in regulating root hair formation in Arabidopsis.

### 3.2. Regulation of Anthocyanin Biosynthesis

As mentioned above, anthocyanin biosynthesis in *Arabidopsis* is another process that is regulated by similar MBW transcription activator complexes. However, instead of activating *GL2*, these complexes regulate anthocyanin biosynthesis by activating the expression of late biosynthesis genes in anthocyanin biosynthesis pathway, including *F3’H*, *DFR*, *ANS*/*LDOX* and *UFGT* [14,15,16,17,18,19,20]. By analyzing *gl2-1D*, an activation-tagged mutant, Wang et al. [26] found that GL2 negatively regulated anthocyanin biosynthesis in *Arabidopsis*. They found that anthocyanin accumulation is reduced in the gain-of-function mutant *gl2-1D*, and elevated in the loss-of-function mutant *gl2-3*. Quantitative RT-PCR results showed that expression of late biosynthesis genes, rather than early biosynthesis genes in anthocyanin biosynthesis pathway was decreased in the *gl2-1D* mutant, and increased in the *gl2-3* mutant. 

Because the expression of the late biosynthesis genes is regulated by the MBW transcriptional activator complexes [16,17,19,73], the authors thus further examined the expression of the MBW component genes in the mutants. They found that the expression of *TT8*, *PAP1*, *PAP2*, *MYB113* and *MYB114* was decreased in the *gl2-1D* mutant, and increased in the *gl2-3* mutant [26]. Whereas chromatin immunoprecipitation assays indicated that at least some of these MBW activator complex component genes are direct targets of GL2 [26]. Consistent with these results, protoplast transient transfection assays showed that when recruited to the promoter region of the *LexA-Gal4:GUS* reporter gene by a fused Gal4 DNA binding domain (GD), GL2 repressed the expression of the reporter gene activated by the transcriptional activator, LD-VP, suggesting that GL2 functions as transcription repressor [26]. The identification of GL2 as a transcription repressor is also consistent with the observation that GL2 was able to directly suppress the expression *RHD6*, *RSL1*, *RSL2*, LRL1 and *LRL2* [66].

Single-repeat R3 MYB transcription factors have been found to regulate anthocyanin biosynthesis by blocking the formation of late biosynthesis genes regulating MBW activator complexes [74,75]. The finding that GL2 negatively regulates anthocyanin biosynthesis in *Arabidopsis* by directly repressing the expression of some of the MBW activator complexes genes adds another feed back loop to the control of anthocyanin biosynthesis. This finding also provides further connection between the regulatory network regulating epidermal cell fate determination including trichome initiation and root hair formation, and the one regulating anthocyanin biosynthesis in *Arabidopsis*.

## 4. Challenges and Future Perspectives

More and more evidence suggest that GL2 plays a central role in the regulation of epidermal cell fate determination and anthocyanin biosynthesis. In addition, GL2 is also involved in the regulation of other processes such as hypocotyl stomatal development, seed coat development, seed oil production, and seed coat mucilage biosynthesis [12,22,35,76,77,78]. However, to uncover the functional mechanisms of GL2 in a different process is still a challenge.

Identification of direct target genes may help to reveal the functional mechanisms of GL2. As a transcription factor, it is reasonable to assume that GL2 regulates these processes by regulating its target gene expression. However, so far, only a few genes have been identified as direct target genes of GL2 (Table 1). Based on large-scale gene expression analyses, a large number of genes were found to be regulated downstream of GL2 during epidermal cell fate determination [70,79,80,81]. A combination of transcriptome analysis and ChIP-qPCR may be used to examine if any of these genes are direct targets of GL2. Transcriptome analysis by using DEX-inducible *GL2* transgenic plants may narrow down candidates of GL2’s direct target genes, and ChIP-qPCR assays may be used to examine if any of the candidate genes are direct targets of GL2. On the other hand, ChIP sequencing will enable genome wide identification of transcription factor binding sites, performing a ChIP sequencing using *GL2* transgenic plants will be a good start for the identification of additional target genes of GL2.

Examining how GL2 regulates its target genes will also help to reveal the functional mechanisms of GL2. Consistent with the results that GL2 functions as a transcription repressor in protoplasts transient transfection assays [26], GL2 negatively regulates the expression of several target genes including *CELLULOSE SYNTHASE5* (*CESA5*), *PLDζ1*, *TT8*, *PAP2* and *MYB113* [82,83]. However, the expression of *XYLOGLUCAN ENDOTRANSGLUCOSYLASE17* (*XTH17*) and *MYB23* was reported to be positively regulated by GL2, and both of them have also been identified as direct target genes of GL2 [60,83]. It may be hard to explain how a transcription repressor may activate gene expression. One speculation is that GL2 may interact with other regulators to regulate its target genes. Indeed, it has been shown that activation of MYB23 requires both GL2 and MYB23 [60], and GIR1 and GIR2 have been found to interact with GL2 to regulate root hair formation [72]. However, considering that GIR1 contains EAR repression motif and functioned as transcription repressor [78], it is unlikely that GL2-GIR1/GIR2 may function as transcription activator complex. Therefore, it will be of interesting to identify and characterize other GL2 interacting proteins if any, and to examine if GIR1 and GIR2 are also involved in the regulation of other growth and development processes regulated by GL2. Considering that GIR1 and GIR2 are also interacting with the major transcription co-repressor TOPLESS (TPL) [84], it may also be of great interest to examine whether GL2, GIR1/GIR2 and TPL may form a repressor complex to regulate the expression of their target genes.

MYBL2, a R3-MYB-related protein, also functions as a transcription repressor [85], and a negative regulator of anthocyanin biosynthesis in *Arabidopsis* [85,86]. In addition to interacting with bHLH transcription factors to inhibit the activity of the MBW activator complexes [85], MYBL2 is able to directly regulate the expression of *TT8* [85], one of the direct target genes of GL2. Therefore, it is worthwhile to examine the relationship between GL2 and MYBL2 in the regulation of anthocyanin biosynthesis in *Arabidopsis*.

It should be note that ANTHOCYANINLESS2 (ANL2), an HD-GLABRA2 group HD-ZIP transcription factor, has been shown to regulate anthocyanin biosynthesis in *Arabidopsis* [87]. In contradiction to the negative role of GL2, ANL2 positively regulates anthocyanin biosynthesis [86]. Examining if ANL2 may regulate the expression of the MBW activator complex component genes, and/or if it may be involved in the regulation of cell fate determination, may help to reveal the relationship between ANL2 and GL2. 

Accumulated evidence suggests that anthocyanin biosynthesis in eudicots is controlled by a conserved transcription factor network [17,88]. It will be of great interest to examine if GL2 homologues in other plants may be involved in the regulation of anthocyanin biosynthesis, if so, their role in the regulation of anthocyanin biosynthesis. 

## Figures and Tables

**Table 1:** Confirmed target genes of GL2 and their functions in *Arabidopsis.*

Targets	Functions	References
*MYB23*	Trichome and root hair formation	Khosla et al., 2014 [60]
*XTH17*	Secondary cell wall formation	Tominaga-Wada et al., 2009 [83]
*CESA5*	Secondary cell wall formation	Tominaga-Wada et al., 2009 [83]
*PLDζ1*	Root hair formation	Ohashi et al.,2003 [82]
*MUM4*	Seed oil production	Shi et al., 2011 [78], Western et al., 2004 [76]
*TT8*	Anthocyanin biosynthesis	Wang et al., 2015 [26]
*PAP1*	Anthocyanin biosynthesis	Wang et al., 2015 [26]
*PAP2*	Anthocyanin biosynthesis	Wang et al., 2015 [26]
*MYB113*	Anthocyanin biosynthesis	Wang et al., 2015 [26]
*MYB114*	Anthocyanin biosynthesis	Wang et al., 2015 [26]
*RHD6*	Root hair formation	Lin et al., 2015 [66]
*RSL1*	Root hair formation	Lin et al., 2015 [66]
*RSL2*	Root hair formation	Lin et al., 2015 [66]
*LRL1*	Root hair formation	Lin et al., 2015 [66]
*LRL2*	Root hair formation	Lin et al., 2015 [66]
